# Distinct role of IL-1β in instigating disease in *Sharpin*^cpdm^ mice

**DOI:** 10.1038/srep36634

**Published:** 2016-11-28

**Authors:** Prajwal Gurung, Bhesh Raj Sharma, Thirumala-Devi Kanneganti

**Affiliations:** 1 Department of Immunology, St. Jude Children’s Research Hospital, Memphis, TN, 38105, USA

## Abstract

Mice deficient in SHARPIN (*Sharpin*^cpdm^ mice), a member of linear ubiquitin chain assembly complex (LUBAC), develop severe dermatitis associated with systemic inflammation. Previous studies have demonstrated that components of the TNF-signaling pathway, NLRP3 inflammasome and IL-1R signaling are required to provoke skin inflammation in *Sharpin*^cpdm^ mice. However, whether IL-1α or IL-1β, both of which signals through IL-1R, instigates skin inflammation and systemic disease is not known. Here, we have performed extensive cellular analysis of pre-diseased and diseased *Sharpin*^cpdm^ mice and demonstrated that cellular dysregulation precedes skin inflammation. Furthermore, we demonstrate a specific role for IL-1β, but not IL-1α, in instigating dermatitis in *Sharpin*^cpdm^ mice. Our results altogether demonstrate distinct roles of SHARPIN in initiating systemic inflammation and dermatitis. Furthermore, skin inflammation in *Sharpin*^cpdm^ mice is specifically modulated by IL-1β, highlighting the importance of specific targeted therapies in the IL-1 signaling blockade.

The chronic proliferative dermatitis (cpdm) phenotype was first identified in C57BL/Ka mice that arose as a result of a spontaneous mutation^1^. A single base-pair deletion in exon 1 of the gene encoding Shank-associated RH domain interacting protein (SHARPIN) was later found to be associated with the cpdm phenotype^2^. As a result, these cpdm mice completely lack SHARPIN expression, and are hereafter referred to as *Sharpin*^cpdm^ mice^2^. *Sharpin*^cpdm^ mice develop severe dermatitis, multiorgan inflammation, and immune system dysregulation^2^. Phenotypically, the dermatitis observed in the *Sharpin*^cpdm^ mice is similar to those in several human inflammatory skin diseases like cryopyrin-associated periodic syndrome, familial Mediterranean fever, and neutrophilic dermatoses^3^.

SHARPIN, Heme-oxidized IRP2 ubiquitin ligase 1 homolog (HOIL-1) and HOIL-1-interacting protein (HOIP) assemble the linear ubiquitin assembly chain complex (LUBAC), which regulates signaling pathways by linearly ubiquitinating target proteins^4^,^5^,^6^. Specifically, SHARPIN is a critical regulator of TNF-mediated cell death pathways- apoptosis and necroptosis^4^,^5^,^6^. As such, *Sharpin*^cpdm^ keratinocytes and fibroblasts are highly sensitive to TNF-induced cell death^4^,^5^,^6^. Indeed, TNF-induced keratinocyte cell death has been proposed to be the major cause of dermatitis observed in *Sharpin*^cpdm^ mice, and TNF-deficiency in *Sharpin*^cpdm^ mice completely prevents dermatitis^4^. More recent studies have identified molecules involved in both apoptotic and necroptotic pathways to be required for cell death induced by TNF in *Sharpin*^cpdm^ mice^7^,^8^. Specifically, deficiency in either caspase-8, FADD, RIPK3, or RIPK1 can prevent induction of dermatitis in *Sharpin*^cpdm^ mice^7^,^8^.

The NLRP3 inflammasome has also been reported to play a role in instigating skin inflammation in *Sharpin*^cpdm^ mice^9^. In comparison to *Sharpin*^cpdm^ mice, *Sharpin*^cpdm^ × *Nlrp3*^−⁄−^ and *Sharpin*^cpdm^ × *Casp1*^−⁄−^ × *Casp11*^−⁄−^ mice display a delayed onset of clinical signs of dermatitis^9^. The NLRP3 inflammasome is a critical regulator of IL-1 cytokines, which signals through IL-1R^10^,^11^,^12^,^13^,^14^. Given that IL-1R deficiency delays the progression of dermatitis in *Sharpin*^cpdm^ mice^8^, we sought to examine the contribution of specific IL-1 cytokines (IL-1α and IL-1β, both of which signal via IL-1R) in the progression of dermatitis and systemic immune perturbation. Herein, we extensively characterize and establish parameters of the immune cell dysregulation found in *Sharpin*^cpdm^ mice. Our data herein demonstrate a specific role for IL-1β in promoting dermatitis in *Sharpin*^cpdm^ mice. Interestingly, IL-1β is dispensable for immune cell dysregulation. Thus, our results suggest that independent mechanisms regulate dermatitis and cellular dysregulation in *Sharpin*^cpdm^ mice. Moreover, IL-1α is completely dispensable for disease progression in *Sharpin*^cpdm^ mice. Altogether, our study further highlights a specific role of IL-1β in provoking dermatitis in *Sharpin*^cpdm^ mice and provides additional evidence advocating the use of specific IL-1 targeted therapies in the treatment of inflammatory diseases.

## Results

### *Sharpin* ^cpdm^ mice develop severe dermatitis and systemic inflammation

**Figure 1:**
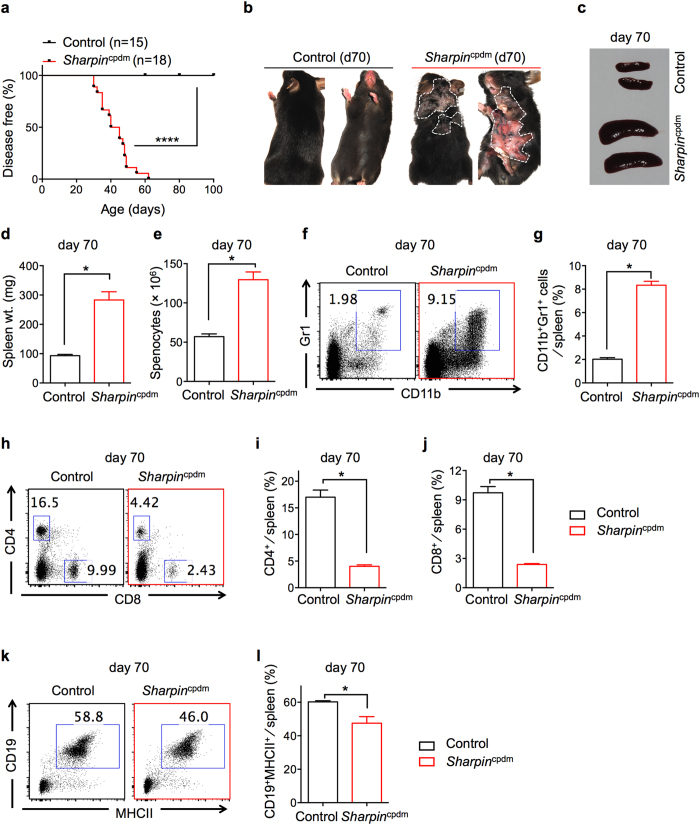
Phenotypic analysis and cellular characterization of diseased *Sharpin*^cpdm^ mice. (**a**) Control (n = 15) and *Sharpin*^cpdm^ (n = 18) mice were followed after weaning and scored for the onset of dermatitis. Mice that showed any sign of skin inflammation were scored as disease-positive and indicated in the disease score curves on that day. (**b**) Representative images of control and *Sharpin*^cpdm^ mice on day 70 post birth, depicting the extent of dermatitis on the dorsal and ventral sides. White dotted line outlines the area of dermatitis in *Sharpin*^cpdm^ mice. (**c–e**) Spleen harvested from control and *Sharpin*^cpdm^ mice on day 70. Representative images (**c**) spleen weight (**d**) and splenocyte count (**e**) of control and *Sharpin*^cpdm^ mice. (**f–l**) Flow cytometry analysis of splenocytes from control and *Sharpin*^cpdm^ mice on day 70. Representative flow plots of CD11b^+^Gr1^+^ neutrophils (**f**) CD4^+^ and CD8^+^ T cells (**h**) and CD19^+^MHCII^+^ B cells (**k**) in the spleen. Cumulative bar graphs representing frequencies of CD11b^+^Gr1^+^ neutrophils (**g**) CD4^+^ T cells (**i**), CD8^+^ T cells (**j**) and CD19^+^MHCII^+^ B cells (**l**) in the spleen. Control, n = 4; *Sharpin*^cpdm^, n = 4 for (**d**, **e**, **g**, **i**, **j, l**). The disease curve in (**a**) was analyzed by log rank (Mantel-Cox) testing. Bar graphs are presented as means ± s.e.m. and are representative of at least three independent experiments. Statistical significance was determined by Mann-Whitney testing, and *P* values less than 0.05 are considered statistically significant. *P < 0.05, ****P < 0.0001.

*Sharpin*^cpdm^ mice develop severe dermatitis that is associated with systemic inflammation. *Sharpin*^cpdm^ mice that were bred in-house developed dermatitis with 100% penetrance at around 30–60 days after birth, with a median age of onset of dermatitis around 42.5 days (Fig. 1a,b). The dermatitis worsened with age, and these mice were eventually euthanized for humane reasons. Further phenotypic analysis demonstrated that 70-day-old diseased *Sharpin*^cpdm^ mice had significantly larger and heavier spleens than those of control mice (Fig. 1c,d). Consistent with the increased spleen size and weight, the total number of splenocytes was also significantly increased in *Sharpin*^cpdm^ mice (Fig. 1e). Analysis of major immune cell populations within the spleen by flow cytometry revealed various abnormalities that were indicative of systemic inflammation in *Sharpin*^cpdm^ mice. The frequency of neutrophils (determined by Gr1^+^CD11b^+^ stained cells) was significantly higher in *Sharpin*^cpdm^ mice than in controls (Fig. 1f,g). In contrast, the frequencies of CD4^+^, CD8^+^, and CD19^+^ cells were significantly lower in the *Sharpin*^cpdm^ spleen than in the control spleen (Fig. 1h–l), which could be a consequence of the increased frequency of neutrophils in the diseased *Sharpin*^cpdm^ mice. As expected with this disease, the frequency of activated antigen-experienced CD8^+^ and CD4^+^ T cells (determined by their surface expression of CD11a^15^,^16^) was also significantly increased in the spleens of *Sharpin*^cpdm^ mice (Extended Data Fig. 1). In accordance with the occurrence of systemic inflammation in *Sharpin*^cpdm^ mice, analysis of neutrophils, T cells, and B cells in peripheral blood leukocytes (PBL) yielded results that were similar to those from the analysis of spleen (Extended Data Fig. 2).

### Immune cellular dysregulation precedes dermatitis in *Sharpin* ^cpdm^ mice

**Figure 2:**
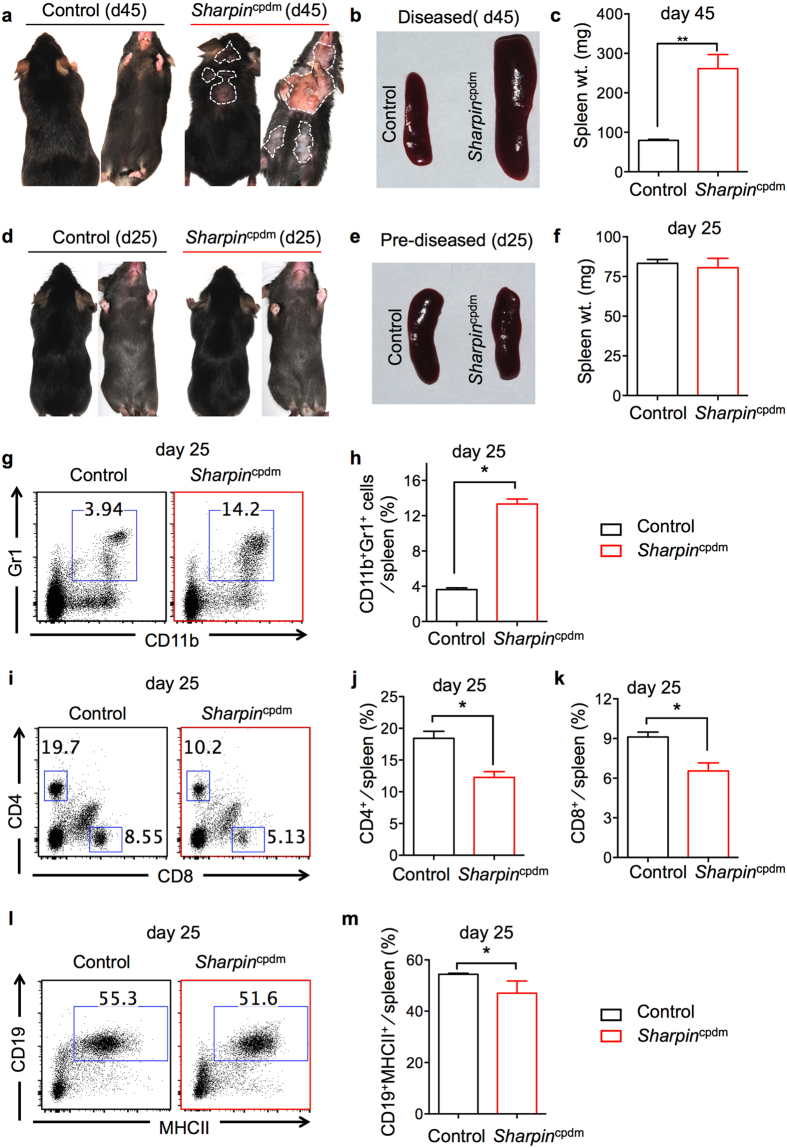
Splenic cell population analysis of pre-diseased *Sharpin*^cpdm^ mice demonstrates cellular dysregulation. Representative images of control and *Sharpin*^cpdm^ mice on day 45 post birth (**a**) and day 25 post birth (**d**), depicting the extent of dermatitis on the dorsal and ventral sides. White dotted line outlines the area of dermatitis in the *Sharpin*^cpdm^ mice. Spleen image (**b,e**) and weight (**c,f**) of control and *Sharpin*^cpdm^ mice on indicated days. (**g–m**) Flow cytometry analysis of splenocytes from control and *Sharpin*^cpdm^ mice on day 25. Representative flow plots of CD11b^+^Gr1^+^ neutrophils (**g**), CD4^+^ and CD8^+^ T cells (**i**), and CD19^+^MHCII^+^ B cells (**l**) in the spleen. Cumulative bar graphs representing frequencies of CD11b^+^Gr1^+^ neutrophils (**h**), CD4^+^ T cells (**j**), CD8^+^ T cells (**k**), and CD19^+^MHCII^+^ B cells (**m**) in the spleen. Control, n = 7; *Sharpin*^cpdm^, n = 6 for (**c**). Control, n = 4; *Sharpin*^cpdm^, n = 4 for **h**, **j**, **k**, and **m**. Bar graphs are presented as means ± s.e.m. and are representative of at least two independent experiments. Statistical significance was determined by Mann-Whitney testing, and *P* values less than 0.05 are considered statistically significant. *P < 0.05.

Although dermatitis and systemic inflammation (associated with splenomegaly and neutrophilia) are two major phenotypes that are observed in diseased *Sharpin*^cpdm^ mice, it is not clear whether systemic inflammation is a consequence of severe dermatitis. Because SHARPIN has distinct roles in different cell types, we examined earlier time points to evaluate the link between dermatitis and systemic inflammation. *Sharpin*^cpdm^ mice developed dermatitis and had significantly increased spleen size and weight at 45 days of age, prompting us to examine earlier time points (Fig. 2a–c). Phenotypic analysis of *Sharpin*^cpdm^ mice showed no signs of dermatitis at 25 days of age (Fig. 2d). Furthermore, the spleen size and weight of *Sharpin*^cpdm^ and control mice were similar, suggesting that no systemic inflammation had occurred by day 25 (Fig. 2e,f).

Interestingly, neutrophil frequency in the spleen was significantly greater in the 25-day-old pre-diseased *Sharpin*^cpdm^ mice than in control mice (Fig. 2g,h). Peripheral blood lymphocytes (PBLs) from pre-diseased *Sharpin*^cpdm^ mice had a similar increase in the frequency of neutrophils (Extended Data Fig. 3a,b). Further, flow cytometry analyses of T- and B-cell populations in the spleen and PBLs revealed that lymphocytes were similarly dysregulated (in comparison with Fig. 1) in 25-day-old pre-diseased *Sharpin*^cpdm^ mice. Both the frequencies of T- and B-cell populations were significantly lower in the spleen and PBLs of 25-day-old *Sharpin*^cpdm^ mice than in those of control mice (Fig. 2i–m and Extended Data Fig. 3c–g). When CD8^+^ and CD4^+^ T cells were further analyzed for activation, as in Fig. 1, we observed increased frequencies of activated CD8^+^ T cells (increased CD11a expression) in both the spleen and PBLs of pre-diseased *Sharpin*^cpdm^ mice compared to control mice (Extended Data Fig. 3h,i and Extended Data Fig. 4a,b). However, the proportion of CD11a^hi^-expressing CD4^+^ T cells in the spleen and PBLs was similar between 25-day-old *Sharpin*^cpdm^ mice and controls (Extended Data Fig. 3j,k and Extended Data Fig. 4c,d).

**Figure 3:**
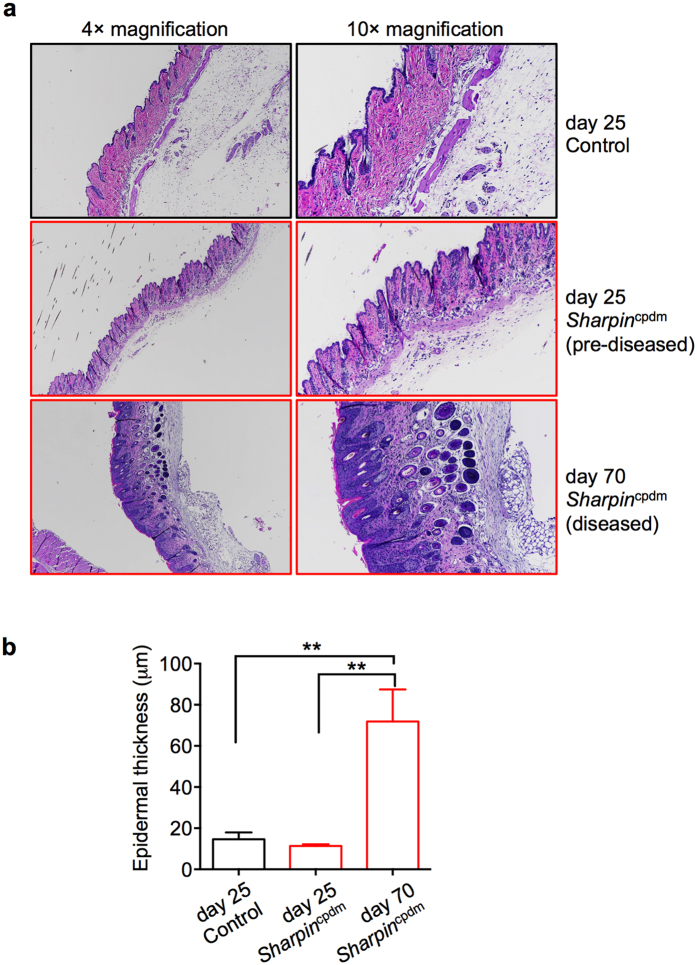
Histological analysis of skin sections from pre-diseased and diseased *Sharpin*^cpdm^ mice. (**a**) Representative hematoxylin and eosin (H&E) images of control, pre-diseased (d25) and diseased (d70) *Sharpin*^cpdm^ mice. (**b**) Epidermal thickness of skin sections from control, pre-diseased (d25) and diseased (d70) *Sharpin*^cpdm^ mice. The epidermis of the H&E sections were measured using FIJI Image J open source software. For each section, four independent measurements were taken and averaged to get a measurement of epidermis thickening. n = 4 for control, n = 4 for pre-diseased and n = 4 for diseased *Sharpin*^cpdm^ groups. Statistical significance was determined by Mann-Whitney testing, and *P* values less than 0.05 are considered statistically significant. *P < 0.05.

To investigate whether immune cell dysregulation precedes skin inflammation, we directly analyzed the skin sections from 25-day-old and 70-day-old *Sharpin*^cpdm^ mice by histology. As ascertained from the H&E stained sections, the epidermal thickness of d25-old *Sharpin*^cpdm^ skin was similar to the skin of control mice, although the 25-day-old *Sharpin*^cpdm^ skin had slightly increased cellular infiltration in the dermis (Fig. 3a). In contrast, the 70-day-old *Sharpin*^cpdm^ skin was visibly inflamed with significantly increased epidermal thickening and cellular infiltrates (Fig. 3a,b). Altogether, these data suggest that systemic dysregulation of immune cells (i.e. increase in neutrophils and activated T cells) precedes dermatitis. However, a previously published study in which *Sharpin*^cpdm^ bone marrow cells were transferred to lethally irradiated WT recipients (*Sharpin*^cpdm^ ≫ WT chimeras) showed that these chimeras did not develop dermatitis, suggesting that dysregulated *Sharpin*^cpdm^ immune cells do not provoke skin inflammation^8^. We independently confirmed these results and found that *Sharpin*^cpdm^ ≫ WT chimeras do not develop dermatitis (Extended Data Fig. 5a). However, PBL analysis of the immune cell population from *Sharpin*^cpdm^ ≫ WT chimeras demonstrated that immune cells have activated T cells and increased neutrophils when compared to WT ≫ WT chimeras (Extended Data Fig. 5b–i). Thus, the immune cell dysregulation and dermatitis observed in *Sharpin*^cpdm^ mice is independent of each other and due to distinct roles of SHARPIN in immune cells and non-hematopoietic cells.

### IL-1R deficiency delays progression of disease in *Sharpin* ^cpdm^ mice

**Figure 4:**
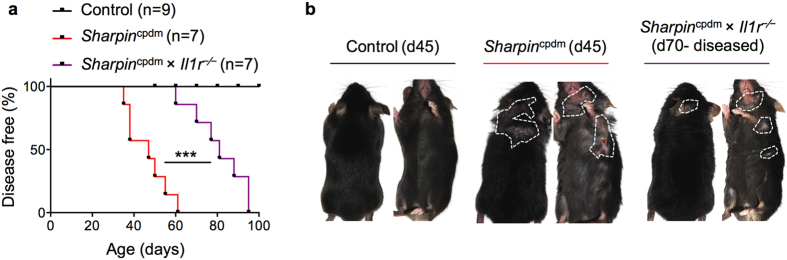
IL-1R deficiency in *Sharpin*^cpdm^ mice delays the development of skin disease. (**a**) Control (n = 9), *Sharpin*^cpdm^ (n = 7), and *Sharpin*^cpdm^ × *Il1r*^−⁄−^ (n = 7) mice were followed after weaning and scored for the onset of dermatitis. Mice that showed any sign of skin inflammation were scored as disease-positive and indicated in the disease score curves on that day. (**b**) Representative images of control and *Sharpin*^cpdm^ mice on day 45 and *Sharpin*^cpdm^ × *Il1r*^−⁄−^ mice on day 70 post birth, depicting the extent of dermatitis on the dorsal and ventral sides. White dotted line outlines the area of dermatitis in *Sharpin*^cpdm^ mice. The disease curve in (**a**) was analyzed by log rank (Mantel-Cox) testing. ***P < 0.001.

The NLRP3 inflammasome is important in regulating disease progression (dermatitis) in *Sharpin*^cpdm^ mice. Specifically, mice deficient in either NLRP3, caspase-1, or caspase-11 demonstrate a delay in progression of disease by about 15–30 days^9^. The NLRP3 inflammasome is a critical regulator of pleiotropic IL-1 cytokines, and cell death termed pyroptosis. Concurrently, mice deficient in IL-1R also delay the onset of dermatitis^8^. To corroborate whether IL-1R deficiency protects *Sharpin*^cpdm^ mice from progression to dermatitis, we generated *Sharpin*^cpdm^ × *Il1r*^−⁄−^ mice and monitored the development of dermatitis in this mice. The disease progression curves of *Sharpin*^cpdm^ and *Sharpin*^cpdm^ × *Il1r*^−⁄−^ mice demonstrated that IL-1R deficiency delayed the progression of disease by 21–30 days (Fig. 4a,b). Overall, the median age of onset of dermatitis for *Sharpin*^cpdm^ mice was 47 days compared to 81 days for *Sharpin*^cpdm^ × *Il1r*^−⁄−^ mice. However, it should be noted that IL-1R deficiency did not provide complete protection and, ultimately, 100% of *Sharpin*^cpdm^ × *Il1r*^−⁄−^ mice developed dermatitis (Fig. 4a).

### IL-1α does not regulate disease progression in *Sharpin* ^cpdm^ mice

**Figure 5:**
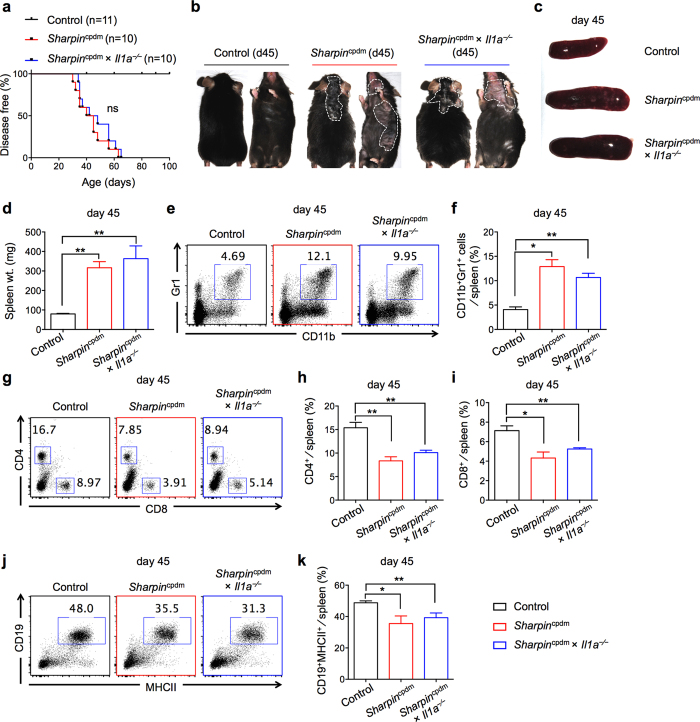
IL-1α is dispensable for induction of disease in *Sharpin*^cpdm^ mice. (**a**) Control (n = 11), *Sharpin*^cpdm^ (n = 10), and *Sharpin*^cpdm^ × *Il1a*^−⁄−^ (n = 10) mice were followed after weaning and scored for the onset of dermatitis. Mice that showed any sign of skin inflammation were scored as disease-positive and indicated in the disease score curves on that day. (**b**) Representative images of control, *Sharpin*^cpdm^, and *Sharpin*^cpdm^ × *Il1a*^−⁄−^ mice on day 45 post birth, depicting the extent of dermatitis on the dorsal and ventral sides. White dotted line outlines the area of dermatitis in *Sharpin*^cpdm^ and *Sharpin*^cpdm^ × *Il1a*^−⁄−^ mice. (**c–d**) Spleen harvested from control, *Sharpin*^cpdm^ and *Sharpin*^cpdm^ × *Il1a*^−⁄−^ mice on day 45. Representative images (**c**) and spleen weight (**d**) of control, *Sharpin*^cpdm^, and *Sharpin*^cpdm^ × *Il1a*^−⁄−^ mice. (**e–k)** Flow cytometry analysis of splenocytes from control, *Sharpin*^cpdm^, and *Sharpin*^cpdm^ × *Il1a*^−⁄−^ on day 45. Representative flow plots of CD11b^+^Gr1^+^ neutrophils (**e**) CD4^+^ and CD8^+^ T cells (**g**) and CD19^+^MHCII^+^ B cells (**j**) in the spleen. Cumulative bar graphs representing frequencies of CD11b^+^Gr1^+^ neutrophils (**f**) CD4^+^ T cells (**h**) CD8^+^ T cells (**i**) and CD19^+^MHCII^+^ B cells (**k**) in the spleen. Control, n = 6; *Sharpin*^cpdm^, n = 6; *Sharpin*^cpdm^ × *Il1a*^−⁄−^, n = 4 for **d**, **f**, **h**, **i, and k**. Disease curve in (**a**) was analyzed by log rank (Mantel-Cox) testing. Bar graphs are presented as means ± s.e.m. Statistical significance between groups was determined by one-way ANOVA followed Dunnett’s multiple comparisons testing, and *P* values less than 0.05 are considered statistically significant. ns = not significant, *P < 0.05, **P < 0.01.

IL-1α is highly expressed in keratinocytes and secreted by cells following cell death. Keratinocyte-specific deletion of TNF and TNFR (signaling molecules involved in apoptotic and necrotic cell death) prevents induction of disease and dermatitis in *Sharpin*^cpdm^ mice^4^,^7^,^8^. Moreover, *Sharpin*^cpdm^ mice also deficient in RIPK1 kinase activity (*Sharpin*^cpdm^ × *Ripk1*^K45A^ mice) are completely protected from disease induction, further demonstrating the importance of cell death pathways^17^. To determine whether IL-1α promotes dermatitis in *Sharpin*^cpdm^ mice, we generated *Sharpin*^cpdm^ × *Il1a*^−⁄−^ mice and observed these mice for signs of disease. *Sharpin*^cpdm^ × *Il1a*^−⁄−^ mice developed dermatitis as early as 30 days after birth, and 100% of these mice developed dermatitis by 70 days with similar kinetics (Fig. 5a). The median age of onset of dermatitis was 42.5 days for *Sharpin*^cpdm^ and 45 days for *Sharpin*^cpdm^ × *Il1a*^−⁄−^ mice (Fig. 5a). *Sharpin*^cpdm^ and *Sharpin*^cpdm^ × *Il1a*^−⁄−^ mice also showed a similar extent of dermatitis and splenomegaly at day 45 post birth (Fig. 5b–d). Histological analysis of skin sections from *Sharpin*^cpdm^ and *Sharpin*^cpdm^ × *Il1a*^−⁄−^ mice showed that both groups had significantly increased cellular infiltration and epidermal thickening when compared to WT controls (Extended data Fig. 6).

Although IL-1α did not provide any protection from dermatitis, we speculated that IL-1α deficiency would rescue the cellular dysregulation observed in *Sharpin*^cpdm^ mice. Analysis of the splenic population for neutrophils, T cells, and B cells revealed dysregulation in *Sharpin*^cpdm^ × *Il1a*^−⁄−^ mice similar to that seen in *Sharpin*^cpdm^ mice (Fig. 5e–k). Specifically, the frequency of Gr1^+^CD11b^+^ neutrophils was significantly increased (Fig. 5e,f), while CD4^+^ T cells (Fig. 5g,h), CD8^+^ T cells (Fig. 5g,i), and CD19^+^ B cells (Fig. 5j,k) were significantly decreased in *Sharpin*^cpdm^ × *Il1a*^−⁄−^ mice, similar to *Sharpin*^cpdm^ mice. Furthermore, the increased activation of CD4^+^ and CD8^+^ T cells observed in *Sharpin*^cpdm^ mice was also observed in *Sharpin*^cpdm^ × *Il1a*^−⁄−^ mice (Extended Data Fig. 7). Similar cellular dysregulation also persisted in the PBLs of *Sharpin*^cpdm^ × *Il1a*^−⁄−^ mice (Extended Data Fig. 8). Altogether, these data suggest that IL-1α is dispensable for induction of dermatitis and cellular dysregulation in *Sharpin*^cpdm^ mice.

### IL-1β deficiency delays the onset of dermatitis in *Sharpin* ^cpdm^ mice

**Figure 6:**
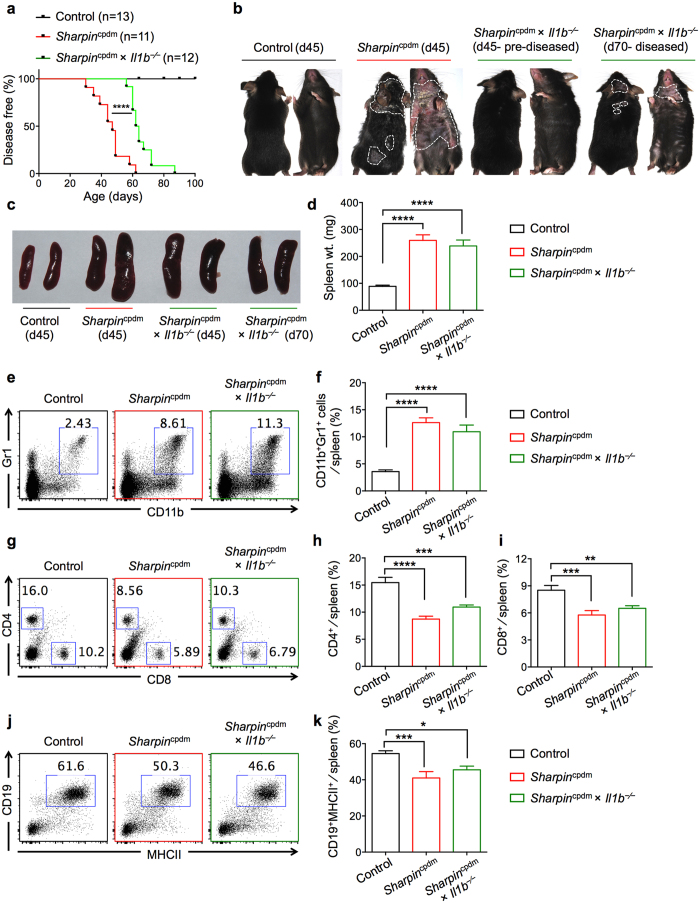
IL-1β deficiency delays the onset of dermatitis in *Sharpin*^cpdm^ mice. (**a**) Control (n = 13), *Sharpin*^cpdm^ (n = 11), and *Sharpin*^cpdm^ × *Il1b*^−⁄−^ (n = 12) mice were followed after weaning and scored for the onset of dermatitis. Mice that showed any sign of skin inflammation were scored as disease-positive and indicated in the disease score curves on that day. (**b**) Representative images of control, *Sharpin*^cpdm^, and *Sharpin*^cpdm^ × *Il1b*^−⁄−^ mice on the indicated days, depicting the extent of dermatitis on the dorsal and ventral sides. White dotted line outlines the area of dermatitis in the *Sharpin*^cpdm^ and *Sharpin*^cpdm^ × *Il1b*^−⁄−^ mice. (**c–d**) Spleen harvested from control, *Sharpin*^cpdm^, and *Sharpin*^cpdm^ × *Il1b*^−⁄−^ mice. Representative images (**c**) and spleen weight (**d**) of control, *Sharpin*^cpdm^, and *Sharpin*^cpdm^ × *Il1b*^−⁄−^ mice. (**e–k**) Flow cytometry analysis of splenocytes from control (d45), *Sharpin*^cpdm^ (d45), and *Sharpin*^cpdm^ × *Il1b*^−⁄−^ mice (d45 and d70 combined). Representative flow plots of CD11b^+^Gr1^+^ neutrophils (**e**) CD4^+^ and CD8^+^ T cells (**g**) and CD19^+^MHCII^+^ B cells (**j**) in the spleen. Cumulative bar graphs representing frequencies of CD11b^+^Gr1^+^ neutrophils (**f**) CD4^+^ T cells (**h**) CD8^+^ T cells (**i**), and CD19^+^MHCII^+^ B cells (**k**) in the spleen. Control, n = 13; *Sharpin*^cpdm^, n = 11; *Sharpin*^cpdm^ × *Il1b*^−⁄−^, n = 10 for **d**, **f**, **h**, **i**, and **k**. Disease curve in (**a**) was analyzed by log rank (Mantel-Cox) testing. Bar graphs are presented as means ± s.e.m. Statistical significance between groups was determined by one-way ANOVA followed by Dunnett’s multiple comparisons testing, and *P* values less than 0.05 are considered statistically significant. *P < 0.05, **P < 0.01, ***P < 0.001, ****P < 0.0001.

Given that IL-1α deficiency did not provide protection from dermatitis or systemic inflammation in *Sharpin*^cpdm^ mice (Fig. 5 and Extended Data Figs 7 and 8), we hypothesized that IL-1β (the other IL-1 cytokine that signals through IL-1R) would be involved in the progression of disease in *Sharpin*^cpdm^ mice. As hypothesized, IL-1β deficiency in *Sharpin*^cpdm^ mice significantly delayed the onset of dermatitis (Fig. 6a). Although all *Sharpin*^cpdm^ mice developed dermatitis between 30–70 days, signs of dermatitis in *Sharpin*^cpdm^ × *Il1b*^−⁄−^ mice were not observed until day 60, with all mice showing some signs of skin inflammation by day 90 (Fig. 6a). The median age of onset of dermatitis for *Sharpin*^cpdm^ mice was 48 days compared to 63 days for *Sharpin*^cpdm^ × *Il1b*^−⁄−^ mice (Fig. 6a). *Sharpin*^cpdm^ mice which were 45 days old developed severe dermatitis, whereas *Sharpin*^cpdm^ × *Il1b*^−⁄−^ mice at this age showed no signs of skin inflammation (Fig. 6b). Histological analysis revealed severe inflammation in the skin of 45-day-old *Sharpin*^cpdm^ mice, characterized by epidermal thickening and the presence of inflammatory cells in the dermis. In contrast, skin sections from pre-diseased 45-day-old *Sharpin*^cpdm^ × *Il1b*^−⁄−^ mice showed significantly reduced epidermal thickening and immune cell infiltration in the dermis (Extended Data Fig. 9). However, *Sharpin*^cpdm^ × *Il1b*^−⁄−^ mice eventually developed dermatitis, as shown for 70-day-old *Sharpin*^cpdm^ × *Il1b*^−⁄−^ mice (Fig. 6b). Although the extent of dermatitis might appear milder in *Sharpin*^cpdm^ × *Il1b*^−⁄−^ mice, they eventually develop severe dermatitis similar to that observed in *Sharpin*^cpdm^ mice.

Analysis of the spleen for signs of systemic inflammation showed that *Sharpin*^cpdm^ × *Il1b*^−⁄−^ mice had increased spleen size and weight, similar to those of *Sharpin*^cpdm^ mice (Fig. 6c,d). To determine whether deficiency in IL-1β rescued cellular dysregulation, we examined various cellular populations in the spleen and PBLs. Interestingly, IL-1β deficiency did not rescue defects in the cell population of *Sharpin*^cpdm^ mice. The frequency of neutrophils significantly increased in both the spleen and PBLs of *Sharpin*^cpdm^ and *Sharpin*^cpdm^ × *Il1b*^−⁄−^ mice compared to control mice (Fig. 6e,f and Extended Data Fig. 10a,b). The frequencies of both CD4^+^ and CD8^+^ T-cells were similarly reduced in *Sharpin*^cpdm^ and *Sharpin*^cpdm^ × *Il1b*^−⁄−^ mice when compared to control mice (Fig. 6g–i and Extended Data Fig. 10c–e). Moreover, further analysis of these T cells showed that higher percentages of the T cells were activated and antigen-experienced in *Sharpin*^cpdm^ and *Sharpin*^cpdm^ × *Il1b*^−⁄−^ mice (Extended Data Fig. 10f–i and Extended Data Fig. 11). Lastly, the frequency of CD19^+^ cells was also reduced in *Sharpin*^cpdm^ and *Sharpin*^cpdm^ × *Il1b*^−⁄−^ mice (Fig. 6j,k). These data demonstrate that although IL-1β delays the onset of dermatitis, it does not provide any protection from cellular dysregulation.

## Discussion

The disease observed in SHARPIN-deficient mice is multifactorial and includes severe dermatitis associated with systemic inflammation and immune cell dysregulation^1^,^2^,^18^. Molecules involved in cell death pathways, including TNFR, FADD, and caspase-8, have a critical role in the development of dermatitis in *Sharpin*^cpdm^ mice^7^,^8^. However, whether genetic deletion of these molecules, which protects *Sharpin*^cpdm^ mice from dermatitis^7^,^8^, also prevents systemic inflammation and immune cell dysregulation has not been thoroughly investigated.

Recent studies from two different groups have demonstrated T-cell intrinsic roles for SHARPIN, revealing a requirement for SHARPIN in regulatory T-cell development and function^19^,^20^. Herein, our studies demonstrate that immune cell dysregulation precedes the development of dermatitis in *Sharpin*^cpdm^ mice. However, SHARPIN-deficient immune cells are not sufficient to establish dermatitis when transferred to WT recipients, as the *Sharpin*^cpdm^ ≫ WT chimeras do not develop any signs of dermatitis, even up to 12 months of age^8^. Supporting this notion, T and B cell–deficient *Sharpin*^cpdm^ mice (*Sharpin*^cpdm^ × *Rag*^−⁄−^ mice) develop dermatitis, although with reduced systemic inflammation^21^. Conversely, *Sharpin*^cdpm^ skin sections that are grafted into nude mice (mice deficient in T cells) maintain their inflamed phenotype even at 3 months post transplant^22^. Taken together, it could be proposed that SHARPIN has distinct roles in the initiation of immune cell dysregulation and dermatitis and that skin intrinsic defects drive dermatitis in *Sharpin*^cdpm^ mice. Importantly, although dysregulated immune cells in *Sharpin*^cpdm^ mice might not be necessary to instigate dermatitis, they are involved in the development of systemic inflammation and their potential role in exacerbating dermatitis in *Sharpin*^cdpm^ mice cannot be excluded.

We have previously shown that SHARPIN is a critical regulator of the NLRP3 inflammasome in mouse bone marrow–derived macrophages and dendritic cells^23^. Macrophages and dendritic cells deficient in SHARPIN are unable to activate caspase-1 and produce IL-1β and IL-18 in response to both canonical (LPS + ATP, LPS + nigericin) and non-canonical (*Citrobacter rodentium* infection) activators of the NLRP3 inflammasome^23^. This work was further corroborated by studies showing that HOIL-1, which comprises the LUBAC in the presence of SHARPIN and HOIP^4^,^5^,^6^, is required for activation of the NLRP3 inflammasome in mouse bone marrow–derived macrophages^24^. In contrast, the lack of SHARPIN promotes NLRP3 inflammasome activation and the secretion of IL-1β, IL-1α, and IL-18 in mouse skin tissues^9^. As a result, *Sharpin*^cpdm^ mice deficient in NLRP3 or caspase-1 demonstrate a delayed onset of dermatitis^9^. Altogether, these results point towards specific roles of SHARPIN in myeloid cells and keratinocytes. Further investigations are required to determine the specific nature of SHARPIN functions in different cell types^3^.

The NLRP3 inflammasome regulates the production of IL-1 cytokines and, thus, the IL-1R signaling axis^25^. In line with the proposed role for NLRP3 in instigating dermatitis^9^, IL-1R deficiency also delays the onset of disease in *Sharpin*^cpdm^ mice^8^. Both IL-1α and IL-1β signal through IL-1R^26^. Recent studies from our laboratory have demonstrated that IL-1β and IL-1α play specific roles in mediating distinct inflammatory diseases^27^,^28^,^29^,^30^. Although IL-1β promotes an inflammatory bone disorder associated with a mouse model of osteomyelitis^27^,^29^, IL-1α is specifically required to promote dermatitis in a mouse model of neutrophilic dermatoses^30^. The specific nature of IL-1α and IL-1β in regulating inflammatory diseases prompted us to examine the contribution of these cytokines in disease progression in *Sharpin*^cpdm^ mice.

Both IL-1α and IL-1β cytokines are upregulated in the diseased *Sharpin*^cpdm^ mice^9^. Cell death pathways involving TNFR, caspase-8, FADD, RIPK3, and RIPK1 are all involved in the disease progression of *Sharpin*^cpdm^ mice^3^. Because IL-1α is released following cell death and plays an important role in the skin inflammation observed in a mouse model of neutrophilic dermatoses^31^, we proposed that IL-1α would have a major role in driving the disease in *Sharpin*^cpdm^ mice. However, IL-1α deficiency did not provide any protection from dermatitis or systemic inflammation in *Sharpin*^cpdm^ mice. These results argue for careful examination of the cause of inflammatory diseases before prescribing a specific treatment therapy.

Although IL-1β deficiency delayed the progression of disease in *Sharpin*^cpdm^ mice, the disease curves of *Sharpin*^cpdm^ × *Il1b*^−⁄−^ mice (Fig. 6) were slightly accelerated than that of *Sharpin*^cpdm^ × *Il1r*^−⁄−^ mice (Fig. 4). Given that both IL-1α and IL-1β signals through IL-1R, the difference in disease curves of *Sharpin*^cpdm^ × *Il1b*^−⁄−^ and *Sharpin*^cpdm^ × *Il1r*^−⁄−^ mice suggests a possible role for IL-1α as well. Interestingly, IL-1α deficiency does not provide any significant protection from disease instigation in *Sharpin*^cpdm^ mice, suggesting a minor role of IL-1α that is revealed upon genetic ablation of IL-1β. Given our hypothesis, we expect that disease curves of *Sharpin*^cpdm^ × *Il1a*^−⁄−^ × *Il1b*^−⁄−^ mice will be similar to that of *Sharpin*^cpdm^ × *Il1r*^−⁄−^ mice.

Our results herein demonstrate that IL-1β is specifically required for the development of dermatitis, but not cellular dysregulation, in *Sharpin*^cpdm^ mice. These results raise several important points that need to be considered. It is possible that other inflammasome-dependent cytokines and effector molecules downstream of caspase-1 could be involved in cellular dysregulation and/or dermatitis development. Specifically, the role of IL-18 in induction of these diseases in *Sharpin*^cdpm^ mice has not been investigated. However, caspase-1–deficiency in *Sharpin*^cpdm^ mice does not prevent splenomegaly, suggesting that the dysregulation of immune cells might be inflammasome-independent^9^. NLRP3 inflammasome activation and release of IL-1β from the cell are associated with pyroptotic cell death. Specifically, pyroptosis is mediated by cleavage of gasdermin D^32^,^33^. Given that several cell death modalities are critical in driving dermatitis in *Sharpin*^cpdm^ mice^3^, it is important to investigate the role of gasdermin D in this inflammatory disease. It will also be of importance to thoroughly investigate whether cellular dysregulation is rescued in *Sharpin*^cpdm^ mice lacking TNF, TNFR, or RIPK1 kinase activity (all these mice are completely protected from developing dermatitis)^3^.

In conclusion, our study examined various cell populations in both the spleen and PBLs and established cellular parameters that can be used to determine cellular dysregulation in *Sharpin*^cpdm^ mice. Our results demonstrate that cellular dysregulation precedes dermatitis in *Sharpin*^cpdm^ mice; however, dermatitis and cellular dysregulation have distinct immunological underpinnings. Our results further show that the onset of dermatitis is specifically modulated by IL-1β, but not by IL-1α. Interestingly, deficiency of IL-1β does not rescue cellular dysregulation, similar to what has been reported for caspase-1/-11–deficient *Sharpin*^cpdm^ mice. Specific effector molecules that promote cellular dysregulation still elude us, and future studies will be required to completely understand and unravel the molecular mechanisms involved in the instigation of this complex disease. Finally, our study provides further evidence that IL-1β and IL-1α have specific roles in regulating inflammatory diseases and appeal for the use of specific therapeutics in treating IL-1–driven diseases.

## Methods

### Guideline statement

All methods used in this study are in accordance with protocols approved by St. Jude Children’s Research Hospital. All studies and experiments were conducted under guidelines and protocols approved by St. Jude Children’s Research Hospital’s Committee on the Use and Care of Animals.

### Mice

C57BL/6J and *Sharpin*^cpdm^ (stock no: 007599) mice were purchased from The Jackson Laboratory and bred at St. Jude Children’s Research Hospital in a specific pathogen–free animal care facility. *Il1a*^−⁄−^ 34, *Il1b*^−⁄−^ 35, and *Il1r*^−⁄−^ 36 mice have been previously described and were bred with *Sharpin*^cpdm^ mice to generate *Sharpin*^cpdm^ × *Il1a*^−⁄−^, *Sharpin*^cpdm^ × *Il1b*^−⁄−^, and *Sharpin*^cpdm^ × *Il1r*^−⁄−^ crosses. Controls used in all figures include combinations of *Sharpin*^WT^ and *Sharpin*^HT^ mice. *Sharpin*^HT^ mice are completely normal and do not develop any dermatitis or inflammatory disease^7^,^8^. Animal studies were conducted under protocols approved by St. Jude Children’s Research Hospital’s Committee on the Use and Care of Animals.

### Generation of chimeras

To generate *Sharpin*^cpdm^ ≫ WT and WT ≫ WT chimeras, WT recipients were lethally irradiated with 900 rads using the Cesium-137 irradiator. Donor bone marrow cells were harvested from the hind limbs (femur and tibia) of *Sharpin*^cpdm^ and WT mice and single cell suspension of bone marrow cells was made. Approximately 5 × 10^6^ *Sharpin*^cpdm^ and WT bone marrow cells were transferred to the lethally irradiated recipients after 6 hours to generate the respective chimeras. These chimeras were followed for up to 120 days and monitored for any signs of disease and dermatitis.

### Scoring of mice for dermatitis for disease-free curves

*Sharpin*^cpdm^, *Sharpin*^cpdm^ × *Il1a*^−⁄−^, *Sharpin*^cpdm^ × *Il1b*^−⁄−^, and *Sharpin*^cpdm^ × *Il1r*^−⁄−^ mice were examined twice weekly for signs of dermatitis. The mice were scored as diseased on the day that the first signs of dermatitis were observed.

### Spleen and PBL processing, fluorescent antibody staining, and flow cytometry analysis

Spleens were harvested from mice after euthanasia, grinded by using a 3-mL syringe plunger, and passed through a 40-μm filter to generate single-cell suspensions. Splenocytes were then treated with 2 mL of ammonium-chloride-potassium (ACK) lysis buffer for 3 minutes to lyse red blood cells (RBCs). After RBC lysis, splenocytes were re-suspended in FACS buffer (PBS + 0.01% NaN_3 _+ 2% FBS) and stained with appropriate flow cytometry antibodies.

PBLs (100 μL) were removed from mice through the retroorbital sinus by using a capillary tube (Drummond Scientific Company, Cat # 2-000-100) and collected in 1.5-mL centrifuge tubes. ACK lysis buffer (1 mL) was added to the cells for 5 minutes to lyse RBCs. After washing, if RBCs still remained, then the process with the ACK lysis buffer was repeated. Following complete RBC lysis, PBLs were stained with flow cytometry antibodies.

RBC-free, single-cell suspensions from spleen or PBL were stained with FITC anti-CD11a (2D7), PE anti-MHCII (M5/114.15.2), PerCP Cy5.5 anti-CD4 (RM4-5), PerCP Cy5.5 anti-Gr1 (RB6-8C5), APC anti-CD44 (IM7), APC anti-CD19 (6D5), eFluor455 anti-CD8 (53–6.7), and eFluor450 anti-CD11b (M1/70) monoclonal antibodies as previously described^37^. Fluorescently labeled cells were then analyzed by using FACS Calibur (BD Biosciences) and FlowJo software (GraphPad PRISM 6, GraphPad Sofware).

### Images and Histopathology

Mouse images were acquired by using a Canon digital camera. Formalin-preserved skin sections were processed and embedded in paraffin according to standard procedures. Sections (5-μm thick) were stained with hematoxylin and eosin (H&E), and images were acquired using light Nikon widefield light microscope.

### Statistical Analysis

All data are represented as the means ± s.e.m., and all experiments were repeated at least twice.

## Additional Information

**How to cite this article**: Gurung, P. *et al.* Distinct role of IL-1β in instigating disease in *Sharpin*^cpdm^ mice. *Sci. Rep.* **6**, 36634; doi: 10.1038/srep36634 (2016).

**Publisher's note:** Springer Nature remains neutral with regard to jurisdictional claims in published maps and institutional affiliations.

## Supplementary Material

Supplementary Information
