# Supplementary material for: Distinct role of IL-1β in instigating disease in Sharpincpdm mice
Source: Sci Rep. 2016 Nov 28;6:36634. doi: 10.1038/srep36634 (PMC5125001; doi:10.1038/srep36634)

SUPPLEMENTARY INFORMATION

Distinct role of IL-1β in instigating disease in *Sharpin*^{cdpm} *mice*

Prajwal Gurung[1], Bhesh Raj Sharma[1] and Thirumala-Devi Kanneganti [1,*]

[1] Department of Immunology, St. Jude Children's Research Hospital, Memphis, TN, 38105, USA.

*** Correspondence should be addressed to:**

Thirumala-Devi Kanneganti

Department of Immunology, St Jude Children's Research Hospital

MS #351, 570, St. Jude Place, Suite E7004

Memphis TN 38105-2794

Tel: (901) 595-3634; Fax. (901) 595-5766.

E-mail: Thirumala-Devi.Kanneganti@StJude.org.

Running Title: IL-1β provokes skin inflammation.

Keywords: SHARPIN, IL-1β, IL-1α, IL-1R, skin inflammation

Extended Data Figures

Extended Data Figure 1. Frequencies of antigen-experienced activated T cells are significantly increased in the spleens of diseased *Sharpin*^{cpdm} mice. Flow cytometry analysis of splenocytes from control and diseased *Sharpin*^{cpdm} mice on day 70 to examine the status of activated T cells. **a**, Representative flow plots showing the frequency of $CD8^{+}CD11a^{hi}$ T cells in the spleen. **b**, Cumulative bar graphs representing frequencies of $CD8^{+}CD11a^{hi}$ T cells in the spleen. **c**, Representative flow plots showing the frequency of $CD4^{+}CD44^{+}CD11a^{hi}$ T cells in the spleen. **d**, Cumulative bar graphs representing frequencies of $CD8^{+}CD11a^{hi}$ T cells in the spleen. Data represented as means ± s.e.m. Control, n=4; *Sharpin*^{cpdm}, n=4 for bar graphs. Statistical significance between groups was determined by Mann-Whitney test and *P* values less than 0.05 were considered statistically significant. $^{*}P<0.05$.

Extended Data Figure 2. Cellular characterization of control and diseased *Sharpin*^{cpdm} in PBL. Flow cytometry analysis of PBL from control and diseased *Sharpin*^{cpdm} mice on day 70. Representative flow plots of $CD11b^{+}Gr1^{+}$ neutrophils (**a**), $CD19^{+}MHCII^{+}$ B cells (**c**), $CD4^{+}$ and $CD8^{+}$ T cells (**e**), $CD8^{+}CD11a^{hi}$ T cells (**h**), and $CD4^{+}CD44^{+}CD11a^{hi}$ T cells (**j**) in the PBL. Cumulative bar graphs representing frequencies of $CD11b^{+}Gr1^{+}$ neutrophils (**b**), $CD19^{+}MHCII^{+}$ B cells (**d**), $CD4^{+}$ T cells (**f**), $CD8^{+}$ T cells (**g**), $CD8^{+}CD11a^{hi}$ T cells (**i**), and $CD4^{+}CD44^{+}CD11a^{hi}$ T cells (**k**) in the PBL. Control, n=7; *Sharpin*^{cpdm}, n=5 for **b**, **d**, **f**, **g**, **i** and **k**. Bar graphs are presented as means ± s.e.m.. Statistical significance was determined by Mann-Whitney test and *P* values less than 0.05 are considered statistically significant. $^{*}P<0.05$, $^{**}P<0.01$.

Extended Data Figure 3. PBL analysis demonstrates cellular dysregulation in pre-diseased *Sharpin*^{cpdm} mice. Flow cytometry analysis of PBL from control and pre-diseased *Sharpin*^{cpdm} mice on day 25 to examine immune cell dysregulation. Representative flow plots of CD11b^{+}Gr1^{+} neutrophils (**a**), CD19^{+}MHCII^{+} B cells (**c**), CD4^{+} and CD8^{+} T cells (**e**), CD8^{+}CD11a^{hi} T cells (**h**), and CD4^{+}CD44^{+}CD11a^{hi} T cells (**j**) in the PBL. Cumulative bar graphs representing frequencies of CD11b^{+}Gr1^{+} neutrophils (**b**), CD19^{+}MHCII^{+} B cells (**d**), CD4^{+} T cells (**f**), CD8^{+} T cells (**g**), CD8^{+}CD11a^{hi} T cells (**i**), and CD4^{+}CD44^{+}CD11a^{hi} T cells (**k**) in the PBL. Control, n=4; *Sharpin*^{cpdm}, n=4 for **b**, **d**, **f**, **g, i** and **k**. Statistical significance between groups was determined by Mann-Whitney test and *P* values less than 0.05 are considered statistically significant. *$P<0.05$.

Extended Data Figure 4. Frequencies of antigen-experienced activated T cells are significantly increased in the spleen of pre-diseased *Sharpin*^{cpdm} mice. Flow cytometry analysis of splenocytes from control and pre-disaesed *Sharpin*^{cpdm} mice on day 25 to examine the status of activated T cells. **a**, Representative flow plots showing the frequency of CD8^{+}CD11a^{hi} T cells in the spleen. **b**, Cumulative bar graphs representing frequencies of CD8^{+}CD11a^{hi} T cells in the spleen. **c**, Representative flow plots showing the frequency of CD4^{+}CD44^{+}CD11a^{hi} T cells in the spleen. **d**, Cumulative bar graphs representing frequencies of CD8^{+}CD11a^{hi} T cells in the spleen. Control, n=4; *Sharpin*^{cpdm}, n=4 for bar graphs. Statistical significance between groups was determined by Mann-Whitney test and *P* values less than 0.05 are considered statistically significant. *$P<0.05$.

Extended Data Figure 5. *Sharpin*^{cpdm} hematopoietic cells are not sufficient to instigate dermatitis in WT recipients. *Sharpin*^{cpdm} >> WT and WT >> WT chimeras were generated by transferring bone marrow cells from *Sharpin*^{cpdm} and WT mice into

lethally irradiated WT recipients. *Sharpin*^{cpdm} >> WT and WT >> WT chimeras were monitored for signs of dermatitis and disease post chimerism. **a**, Disease free curves for *Sharpin*^{cpdm} >> WT and WT >> WT chimeras. **b-l**, PBL from *Sharpin*^{cpdm} >> WT and WT >> WT chimeras on day 100 to examine immune cell dysregulation. Representative flow plots of CD4^{+} and CD8^{+} T cells (**b**), CD4^{+}CD44^{+}CD11a^{hi} T cells (**e**), CD8^{+}CD11a^{hi} T cells (**g**), CD11b^{+}Gr1^{+} neutrophils (**i**), and CD19^{+}MHCII^{+} B cells (**k**) in the PBL. Cumulative bar graphs representing frequencies of CD4^{+} T cells (**c**), CD8^{+} T cells (**d**), CD4^{+}CD44^{+}CD11a^{hi} T cells (**f**), CD8^{+}CD11a^{hi} T cells (**h**) CD11b^{+}Gr1^{+} neutrophils (**j**), and CD19^{+}MHCII^{+} B cells (**l**) in the PBL. WT >> WT, n=4; *Sharpin*^{cpdm} >> WT, n=5 for **c**, **d**, **f**, **h, j** and **l**. Statistical significance between groups was determined by Mann-Whitney test and *P* values less than 0.05 are considered statistically significant. *$P<0.05$.

Extended Data Figure 6. Histological analysis of skin sections from *Sharpin*^{cpdm} and *Sharpin*^{cpdm} × *Il1a*$^{-/-}$ mice. a, Representative hematoxylin and eosin (H&E) images of control, *Sharpin*^{cpdm} and *Sharpin*^{cpdm} × *Il1a*$^{-/-}$ mice. **b**, Epidermal thickness measured from the skin sections of control, *Sharpin*^{cpdm} and *Sharpin*^{cpdm} × *Il1a*$^{-/-}$ mice. The epidermal thickness was measured using FIJI Image J open source software. n=4 for control, n=4 for *Sharpin*^{cpdm} and n=4 for *Sharpin*^{cpdm} × *Il1a*$^{-/-}$ groups. Statistical significance was determined by Mann-Whitney testing, and *P* values less than 0.05 are considered statistically significant. *$P<0.05$.

Extended Data Figure 7. IL-1α deficiency does not rescue activated T cell phenotype in *Sharpin*^{cpdm} mice. Flow cytometry analysis of splenocytes from control, *Sharpin*^{cpdm} and *Sharpin*^{cpdm} × *Il1a*$^{-/-}$ mice on day 45 to examine the status of activated T cells. **a**, Representative flow plots showing the frequency of CD4^{+}CD44^{+}CD11a^{hi} T cells in the spleen. **b**, Cumulative bar graphs representing frequencies of CD4^{+}CD44^{+}CD11a^{hi}

T cells in the spleen. **c**, Representative flow plots showing the frequency of $CD8^+CD11a^{hi}$ T cells in the spleen. **d**, Cumulative bar graphs representing frequencies of $CD8^+CD11a^{hi}$ T cells in the spleen. Control, n=6; *Sharpin*^{cpdm}, n=6; *Sharpin*^{cpdm} × *Il1a*$^{-/-}$, n=4 for bar graphs. Statistical significance was determined by one-way ANOVA, Dunnett's multiple comparisons test and *P* values less than 0.05 are considered statistically significant. $^{**}P<0.01$.

Extended Data Figure 8. PBL analysis of *Sharpin*^{cpdm} and *Sharpin*^{cpdm} × *Il1a*$^{-/-}$ mice. Flow cytometry analysis of PBL from control, *Sharpin*^{cpdm} and *Sharpin*^{cpdm} × *Il1a*$^{-/-}$ mice on day 45 to examine immune cell dysregulation. Representative flow plots of $CD11b^+Gr1^+$ neutrophils (**a**), $CD4^+$ and $CD8^+$ T cells (**c**), $CD4^+CD11a^{hi}$ T cells (**f**), and $CD8^+CD11a^{hi}$ T cells (**h**) in the PBL. Cumulative bar graphs representing frequencies of $CD11b^+Gr1^+$ neutrophils (**b**), $CD4^+$ T cells (**d**), $CD8^+$ T cells (**e**), $CD8^+CD11a^{hi}$ T cells (**g**), and $CD4^+CD44^+CD11a^{hi}$ T cells (**i**) in the PBL. Control, n=11; *Sharpin*^{cpdm}, n=4; *Sharpin*^{cpdm} × *Il1a*$^{-/-}$, n=4 for **b**, **d**, **e**, **g,** and **i**. Bar graphs are presented as means ± s.e.m. Statistical significance between groups was determined by one-way ANOVA, Dunnett's multiple comparisons test and *P* values less than 0.05 are considered statistically significant. $^{****}P<0.0001$.

Extended Data Figure 9. Histological analysis of skin sections from *Sharpin*^{cpdm} and *Sharpin*^{cpdm} × *Il1b*$^{-/-}$ mice. a, Representative hematoxylin and eosin (H&E) images of control, *Sharpin*^{cpdm} and *Sharpin*^{cpdm} × *Il1b*$^{-/-}$ mice. **b**, Epidermal thickness of skin sections from control, *Sharpin*^{cpdm} and *Sharpin*^{cpdm} × *Il1b*$^{-/-}$ mice. The epidermis of the H&E sections were measured using FIJI Image J open source software. n=4 for control, n=4 for *Sharpin*^{cpdm} and n=4 for *Sharpin*^{cpdm} × *Il1b*$^{-/-}$ groups. Data represented as means

± s.e.m. Statistical significance was determined by Mann-Whitney testing, and *P* values less than 0.05 are considered statistically significant. **P*<0.05, ns=not significant.

Extended Data Figure 10. PBL analysis of *Sharpin*^{cpdm} and *Sharpin*^{cpdm} × *Il1b*$^{-/-}$ mice. Flow cytometry analysis of PBL from control, *Sharpin*^{cpdm} and *Sharpin*^{cpdm} × *Il1b*$^{-/-}$ mice to examine immune cell dysregulation. Representative flow plots of $CD11b^+Gr1^+$ neutrophils (**a**), $CD4^+$ and $CD8^+$ T cells (**c**), $CD4^+$ $CD44^+CD11a^{hi}$ T cells (**f**), and $CD8^+CD11a^{hi}$ T cells (**h**) in the PBL. Cumulative bar graphs representing frequencies of $CD11b^+Gr1^+$ neutrophils (**b**), $CD4^+$ T cells (**d**), $CD8^+$ T cells (**e**), $CD4^+CD44^+CD11a^{hi}$ T cells (**g**), and $CD8^+CD11a^{hi}$ T cells (**i**) in the PBL. Control, n=11; *Sharpin*^{cpdm}, n=9; *Sharpin*^{cpdm} × *Il1b*$^{-/-}$, n=7 for **b**, **d**, **e**, **g,** and **i**. Bar graphs are presented as means ± s.e.m. Statistical significance between groups was determined by one-way ANOVA, Dunnett's multiple comparisons test and *P* values less than 0.05 are considered statistically significant. **P*<0.05, ***P*<0.01, ****P*<0.001, *****P*<0.0001.

Extended Data Figure 11. IL-1β deficiency does not rescue activated T cell phenotype in *Sharpin*^{cpdm} mice. Flow cytometry analysis of splenocytes from control, *Sharpin*^{cpdm} and *Sharpin*^{cpdm} × *Il1b*$^{-/-}$ mice to examine the status of activated T cells. **a**, Representative flow plots showing the frequency of $CD4^+CD44^+CD11a^{hi}$ T cells in the spleen. **b**, Cumulative bar graphs representing frequencies of $CD4^+CD44^+CD11a^{hi}$ T in the spleen. **c**, Representative flow plots showing the frequency of $CD8^+CD11a^{hi}$ T cells in the spleen. **d**, Cumulative bar graphs representing frequencies of $CD8^+CD11a^{hi}$ T in the spleen. Control, n=13; *Sharpin*^{cpdm}, n=11; *Sharpin*^{cpdm} × *Il1b*$^{-/-}$, n=10 for bar graphs. Statistical significance between groups was determined by one-way ANOVA, Dunnett's multiple comparisons test and *P* values less than 0.05 are considered statistically significant. **P*<0.05, ***P*<0.01, ****P*<0.001.

Extended Data Figure 1

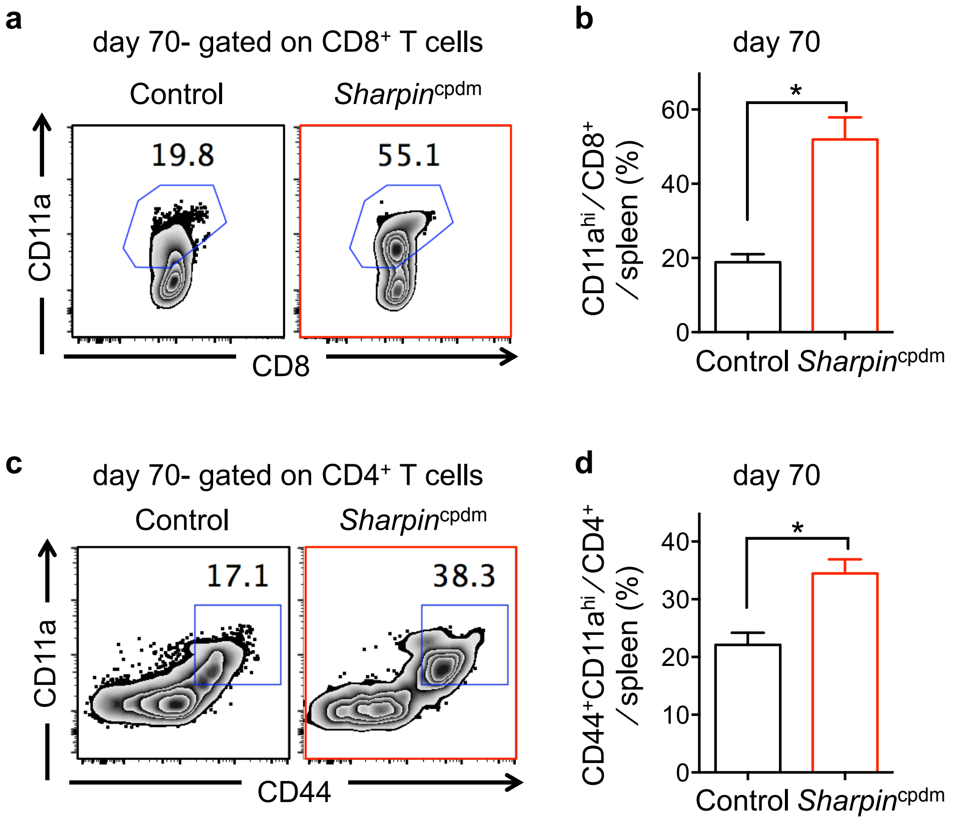

Extended Data Figure 2

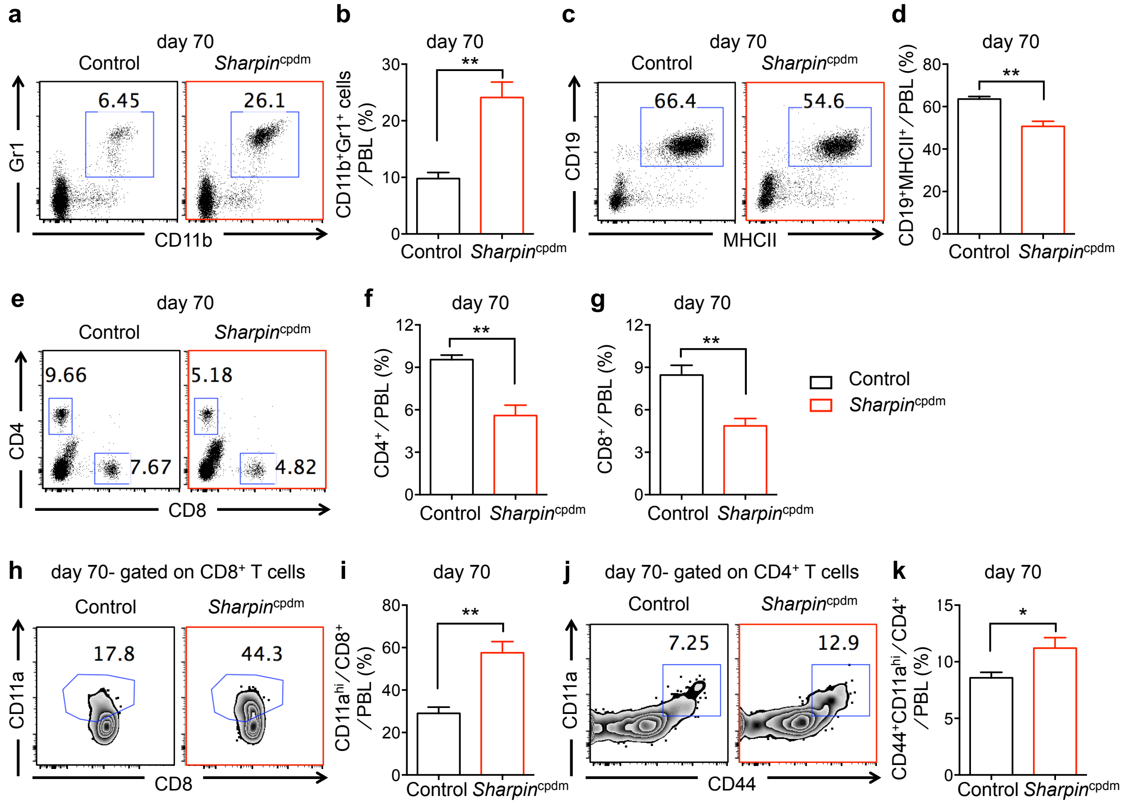

Extended Data Figure 3

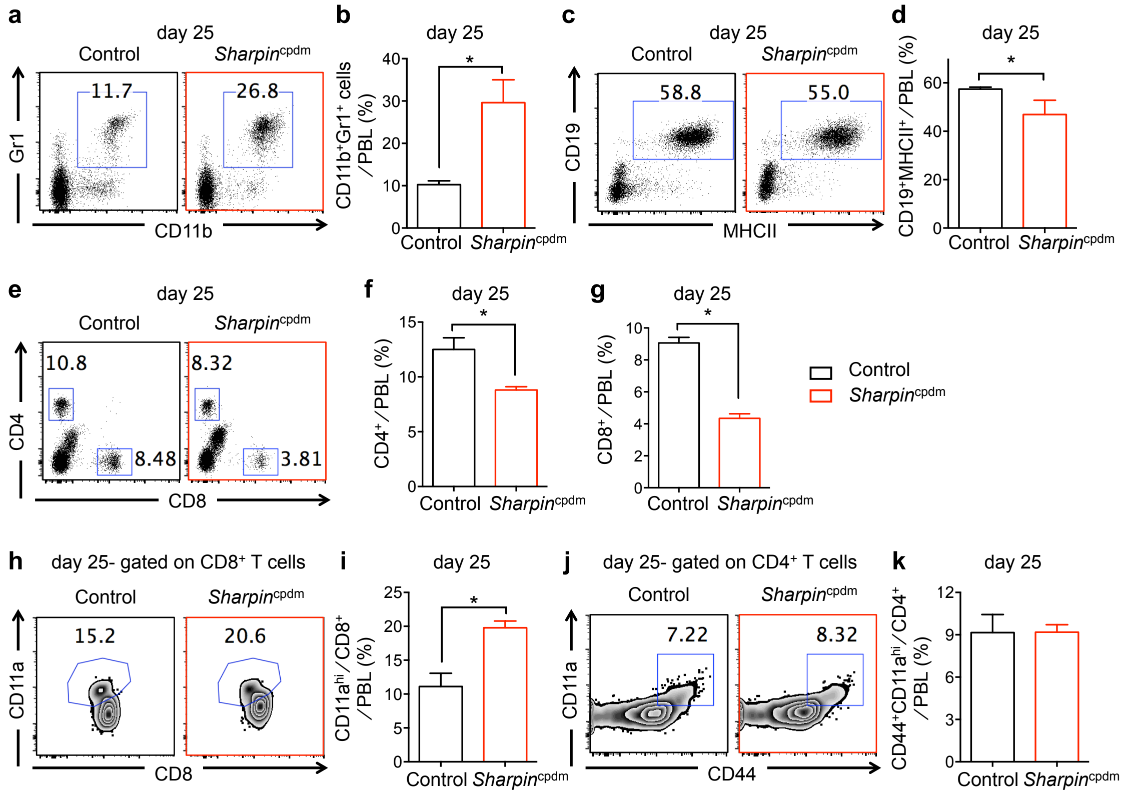

Extended Data Figure 4

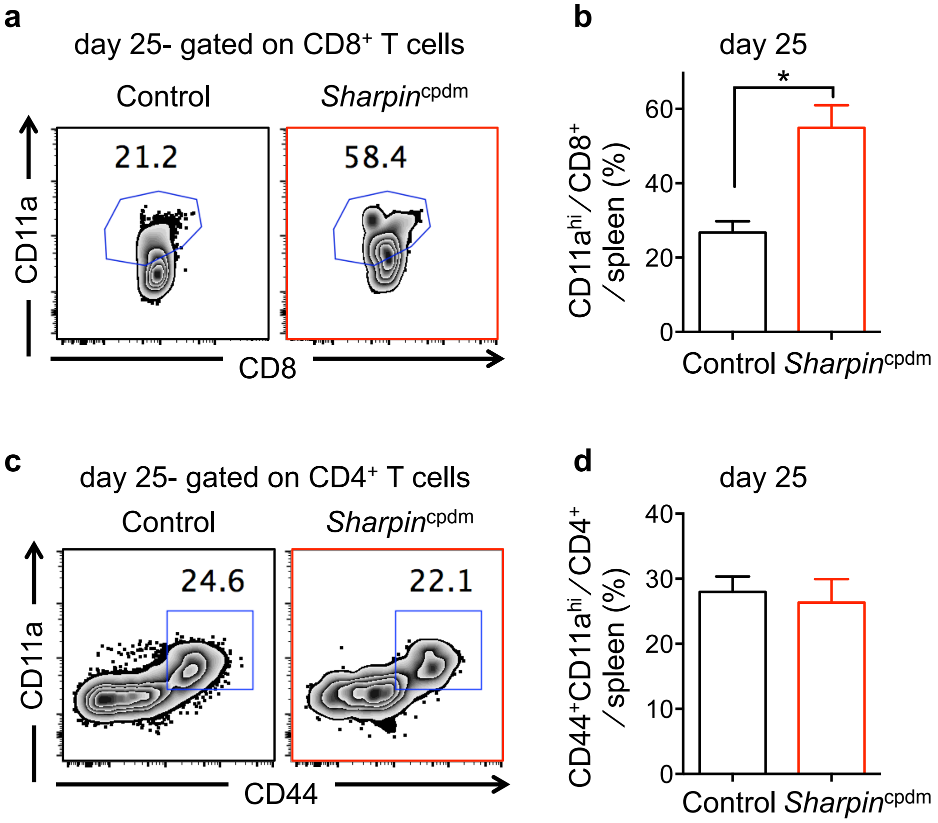

Extended Data Figure 5

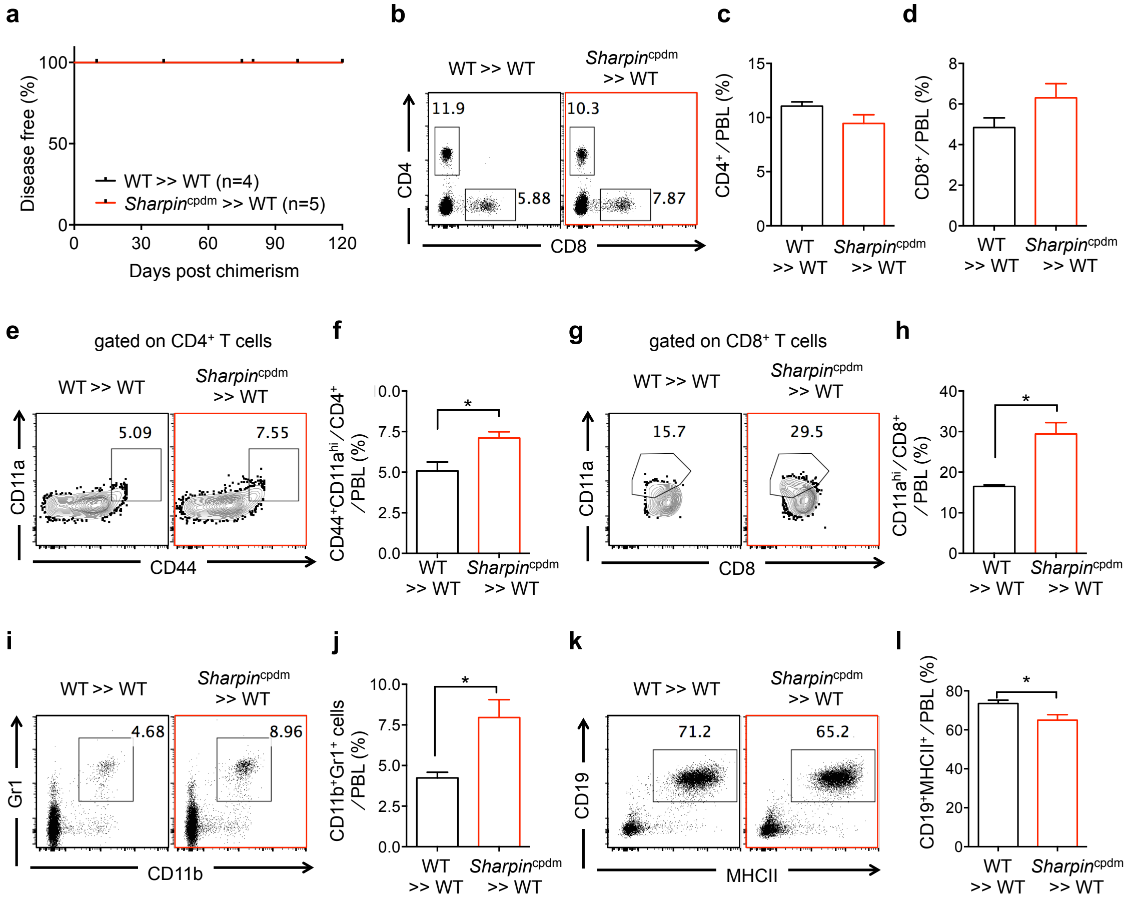

Extended Data Figure 6

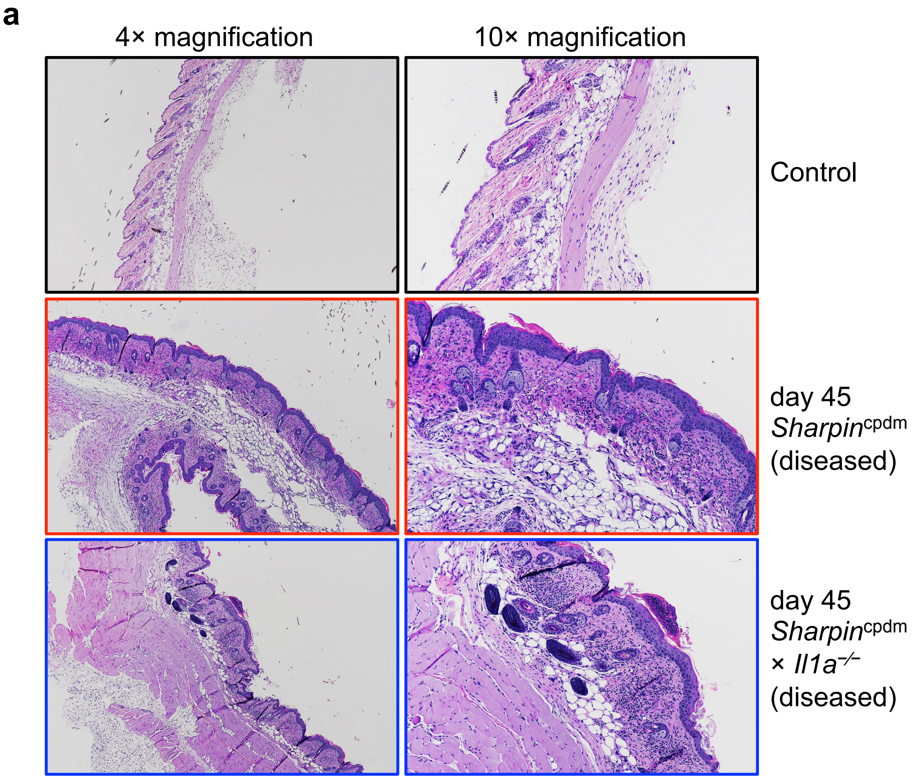

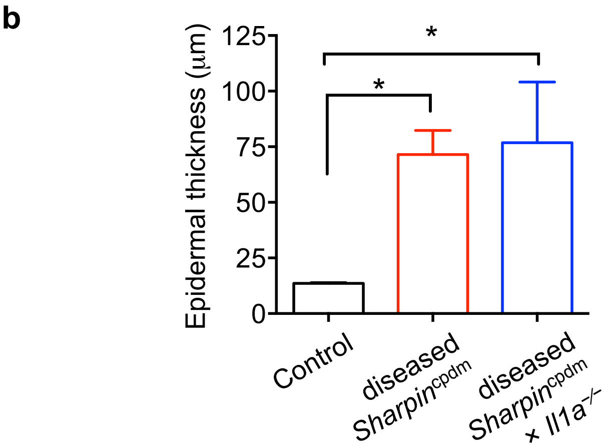

Extended Data Figure 7

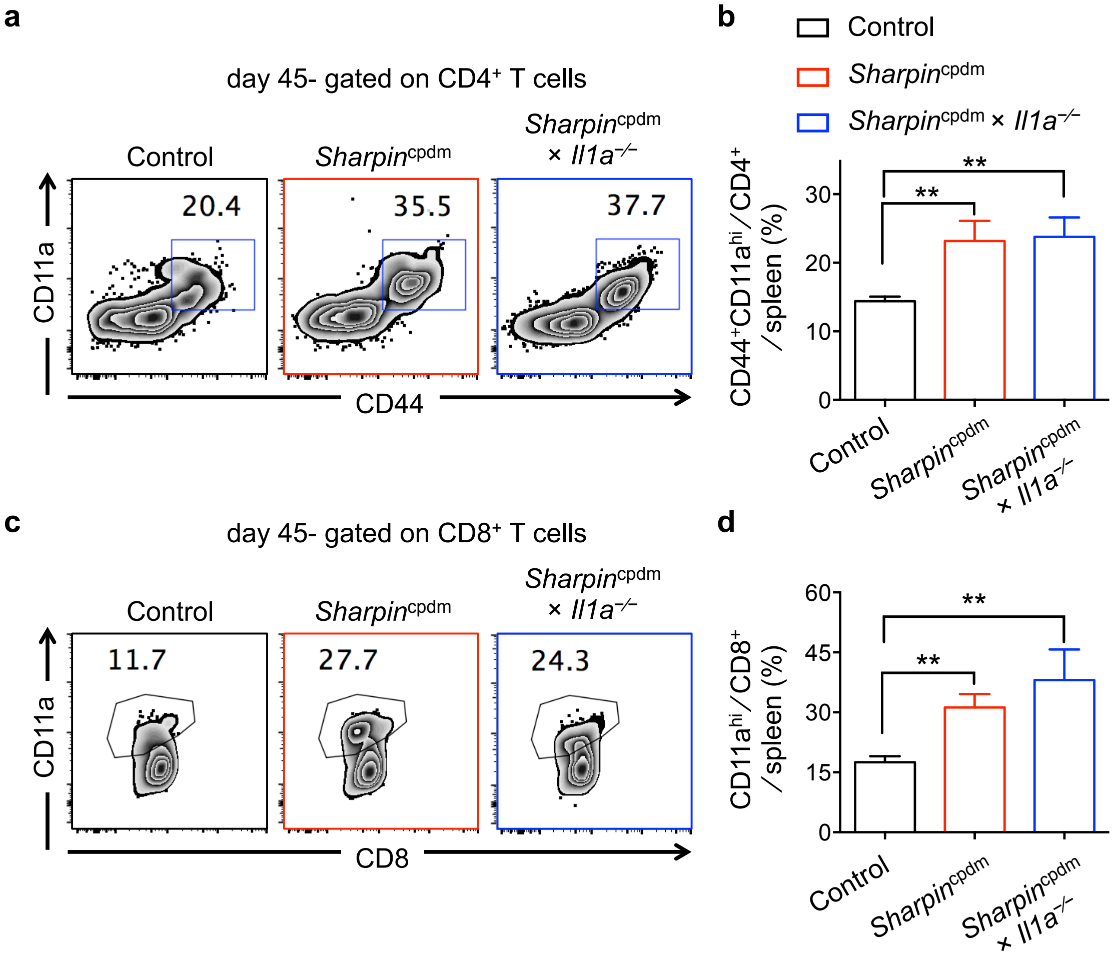

Extended Data Figure 8

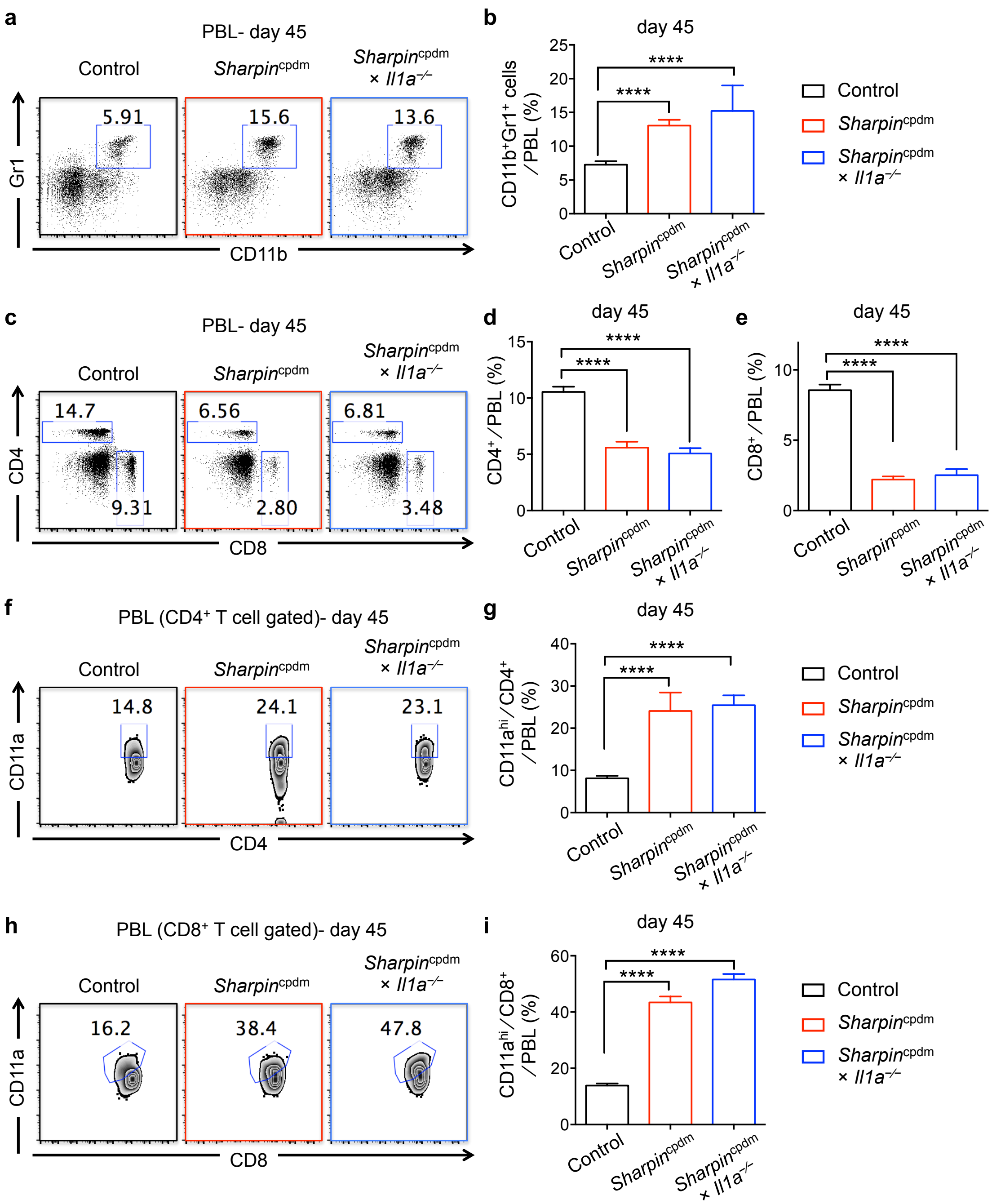

Extended Data Figure 9

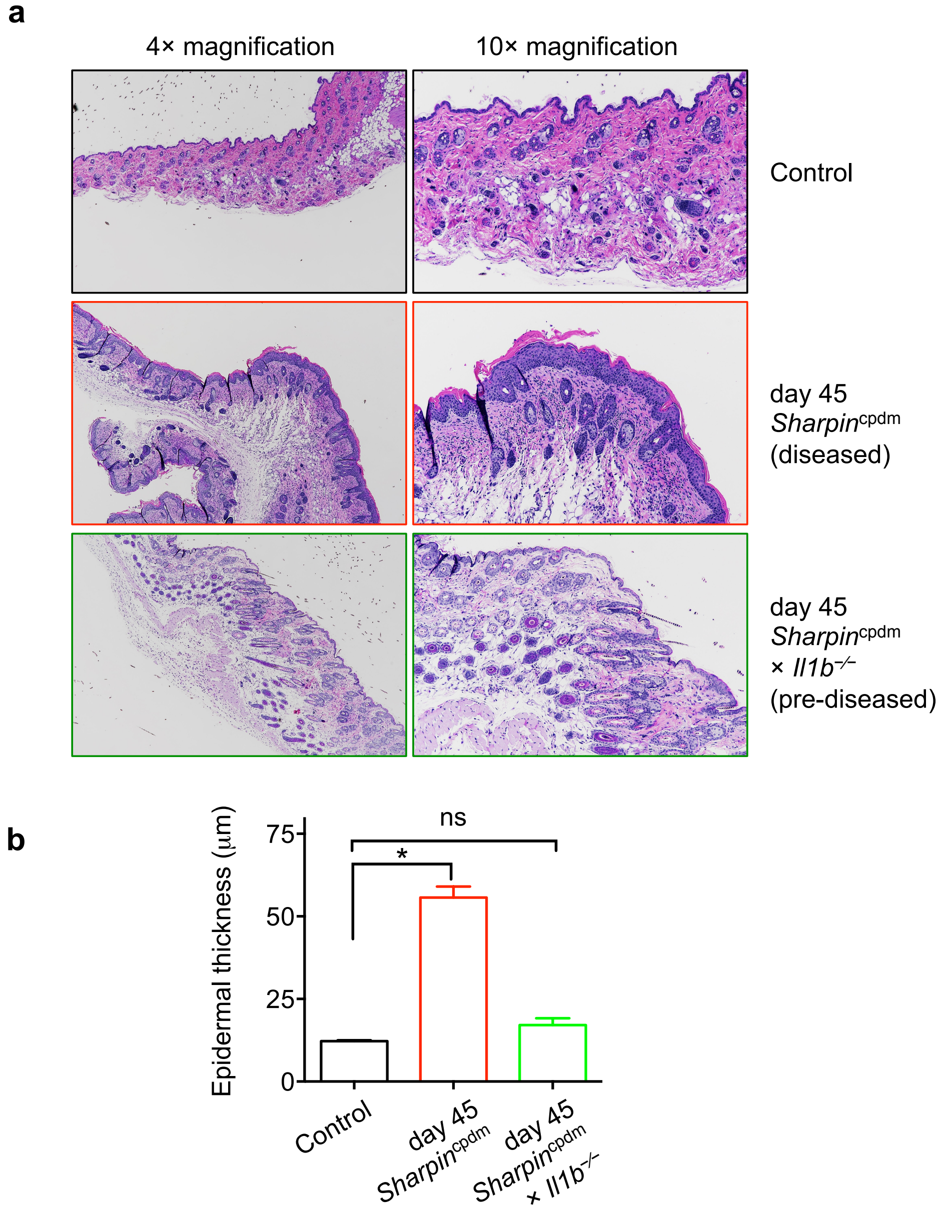

Extended Data Figure 10

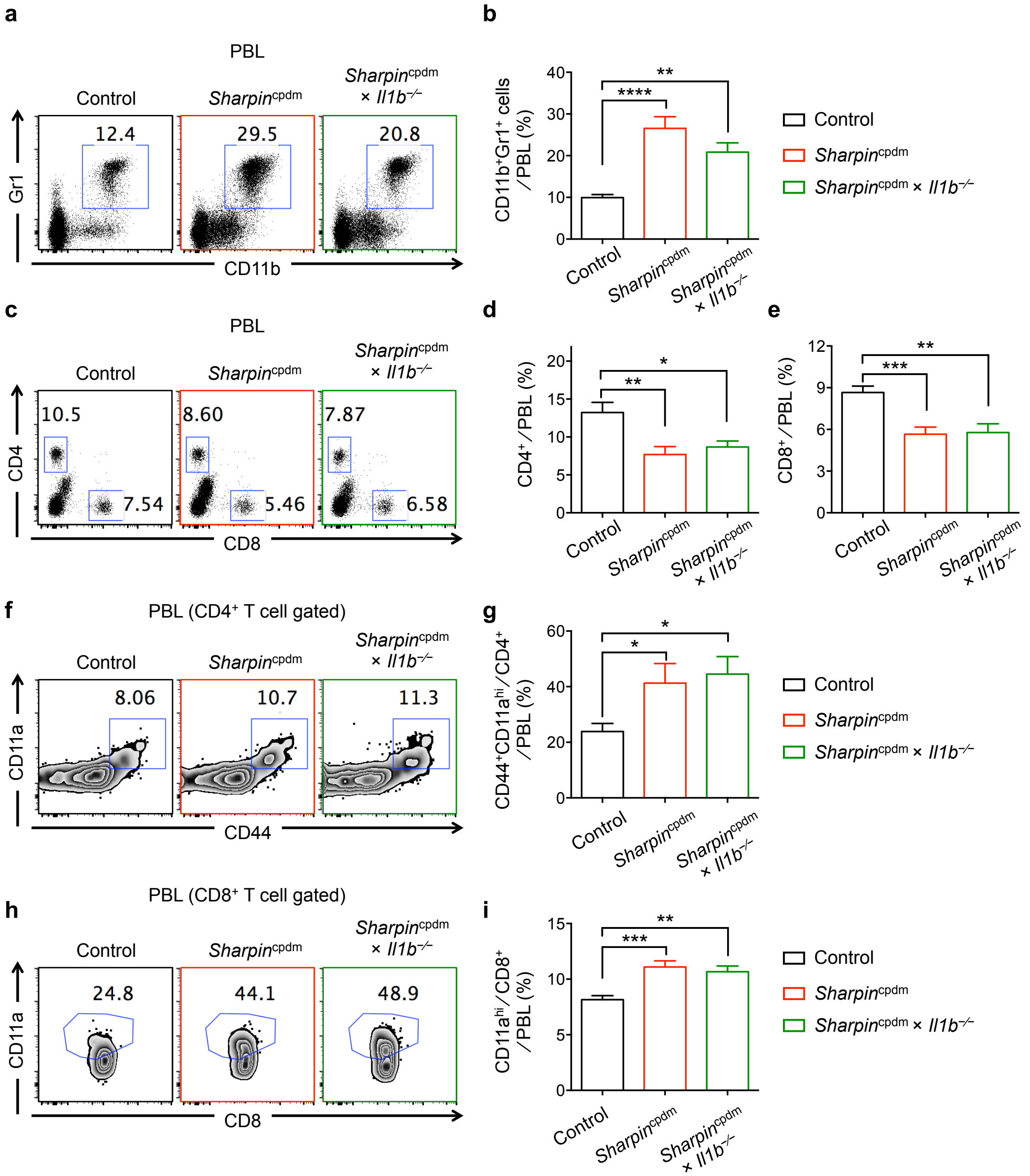

Extended Data Figure 11

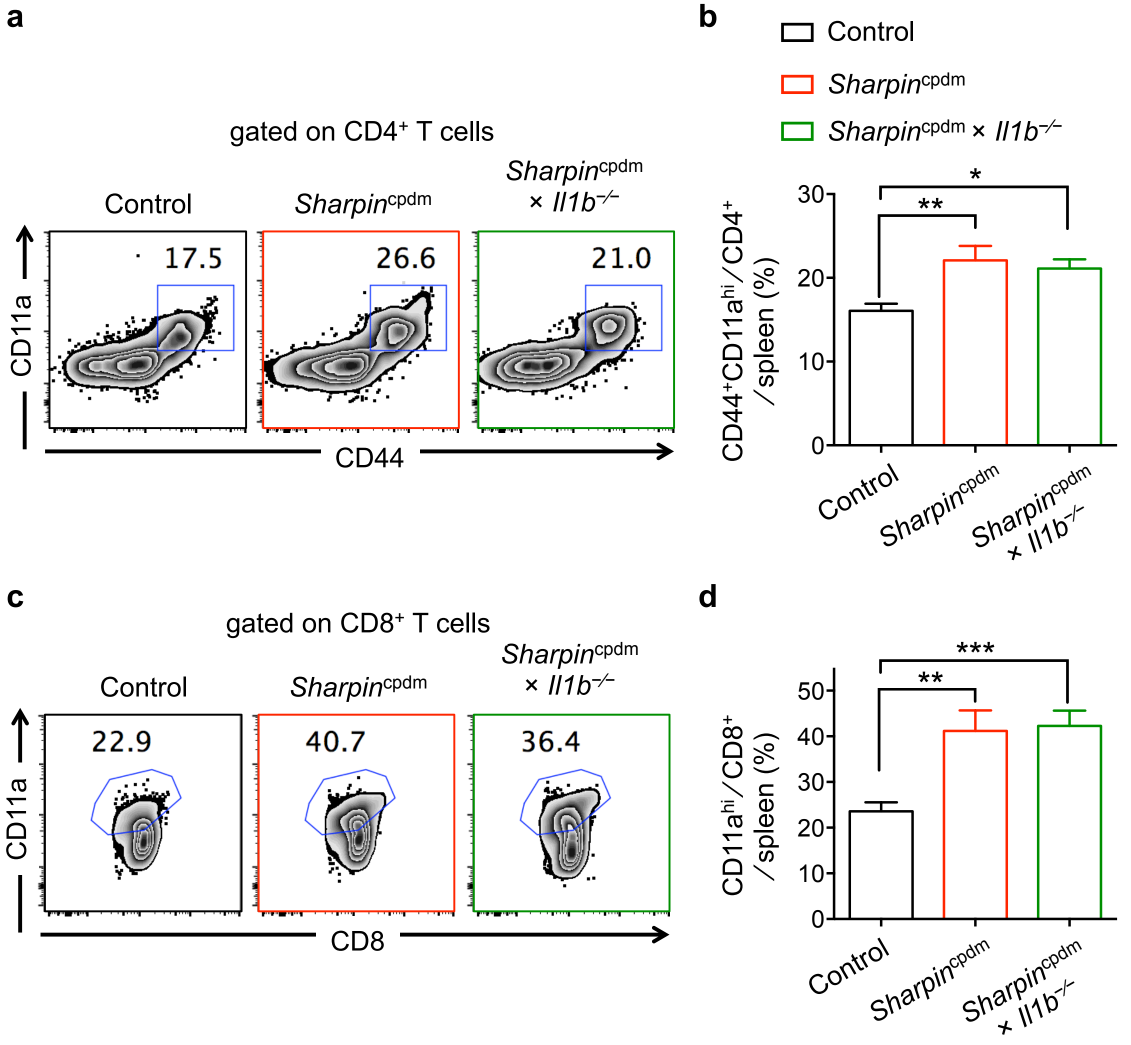

Supplement: Supplementary Information [file srep36634-s1.pdf]
